# Clinical and molecular characteristics of *Klebsiella pneumoniae* infection in a tertiary general hospital of Wuhan, China

**DOI:** 10.1007/s10096-023-04719-1

**Published:** 2023-12-01

**Authors:** Yating Xiang, Hongpan Tian, Qingsong Chen, Jihong Gu, Hongmao Liu, Cuixiang Wang, Yirong Li

**Affiliations:** 1 Department of Clinical Laboratory, Zhongnan Hospital of Wuhan University, Wuhan, People’s Republic of China; 2 Wuhan Research Center for Infectious Diseases and Cancer, Chinese Academy of Medical Sciences, Wuhan, People’s Republic of China; 3 Hubei Engineering Center for Infectious Disease Prevention, Control and Treatment, Wuhan, People’s Republic of China

**Keywords:** CRKP, CSKP, ST11, Virulence genes, *Bla*_KPC-2_, *Bla*_NDM_ like, *Bla*_OXA-48_ like

## Abstract

**Objectives:**

The aim of this study was to investigate the clinical and molecular characteristics of *Klebsiella pneumoniae* infection from a tertiary general hospital in Wuhan, China.

**Methods:**

From December 2019 to August 2022, 311 non-duplicate isolates of *K. pneumoniae* were collected from a tertiary hospital in Wuhan. These comprised 140 carbapenem-resistant *K. pneumoniae* (CRKP) isolates and 171 carbapenem-susceptible *K. pneumoniae* (CSKP) isolates. The clinical characteristics of patients with *K. pneumoniae* infection were retrospectively collected. Polymerase chain reaction (PCR) assays were used to identify the main carbapenem resistance genes, virulence genes and multi-locus sequence typing (MLST) profiles of the isolates, and the *Galleria mellonella* infection model was used to determine their virulence phenotypes.

**Results:**

Independent risk factors for CRKP infection were hypertension, neurological disorders, being admitted to the intensive care unit (ICU) and prior use of antibiotics. Patient with CRKP infection had higher mortality than those with CSKP infection (23.6% vs 14.0%, *P* < 0.05). One hundred and two sequence types (STs) were identified among the *K. pneumoniae* isolates, and the most prevalent ST type was ST11 (112/311, 36.0%). All of the ST11 isolates were CRKP. Among the 112 ST11 isolates, 105 (93.8%) harboured the carbapenem resistance gene *bla*_KPC-2_ (ST11-*KPC-2*), and of these isolates, 78 (74.3%, 78/105) contained all of the four virulence genes, namely *rmpA, rmpA2*, *iroN* and *iucA*, suggesting that these genes were widespread among the isolates responsible for *K. pneumoniae* infections.

**Conclusion:**

In this study, ST11-*KPC-2* was responsible for most of the *K. pneumoniae* infection cases. Carbapenem resistance rather than the co-occurrence of the virulence genes *rmpA*, *rmpA2*, *iroN* and *iucA* was associated with *K. pneumoniae* infection-related mortality during hospitalisation. Furthermore, a high proportion of ST11-*KPC-2* isolates carried all of the four virulence genes.

## Introduction

*Klebsiella pneumoniae* is a gram-negative, nonmotile, encapsulated opportunistic pathogen that generally colonises human mucous membranes, including the nasopharynx and gastrointestinal tract and is ubiquitously found in soils and the healthcare environment such as on the surfaces of medical devices [1, 2]. In immunocompromised patients, infants and older adults, it can cause a variety of infectious diseases, including lower respiratory tract infections, urinary tract infections, bacteraemia and sepsis, and can even lead to infection-related death [3–5]. Furthermore, infection caused by drug-resistant *K. pneumoniae*, especially by carbapenem-resistant *K. pneumoniae* (CRKP), are known to be associated with longer hospital stays, higher medical costs and a higher risk of mortalities than those caused by carbapenem-susceptible *K. pneumoniae* [6].

Carbapenem is regarded as an effective antimicrobial agent for treating serious *K. pneumoniae* infections [7]. However, carbapenem resistance has been found in *K. pneumoniae* isolates in many regions worldwide and has been recognised as a major public health concern due to its rapid transmission [8]. In China, the average resistance rates of *K. pneumoniae* to imipenem and meropenem were 22.6% and 24.2%, respectively, in 2022, which were significantly higher than the rates of 2.9% and 3.0% reported in 2005 (http://www.chinets.com) [9]. *K. pneumoniae* carbapenemase (KPC) and New Delhi metallo-β-lactamase (NDM) encoded by *bla*_KPC_ and *bla*_NDM_, respectively, are the commonest factors responsible for carbapenem resistance in *K. pneumoniae* in most countries worldwide, whereas oxacillinase-48 (OXA-48) is most common in Africa [10–12]. Moreover, co-occurrence of *bla*_KPC-2_ and *bla*_NDM_ in *K. pneumoniae* has been reported in the USA, China and Brazil [13, 14]. A promising new β-lactamase inhibitor combination for treating infections caused by KPC- and NDM-co-producing *K. pneumoniae* is aztreonam-avibactam [15, 16].

Hypervirulent CRKP (hv-CRKP) is a growing public concern due to its high drug resistance and virulence, posing a serious threat to human health [17]. In 2018, in a hospital outbreak of ST11 hv-CRKP in Zhejiang province, China, the fatality rate was 100%. This was found to be attributable to the presence of pLVPK, a virulence plasmid (~ 170 kb long) containing two capsular polysaccharide regulatory genes (*rmpA* and *rmpA2*) and multiple siderophore gene clusters (*iucABCD*/*iutA/iroBCDN*), which imparted concurrent hypervirulence, multidrug resistance and high transmissibility. It was also shown that the occurrence of the virulence genes *rmpA*, *rmpA2*, *iroN* and *iucA* was correlated with the presence of the virulence plasmid pLVPK [18–20]. Furthermore, an increasing occurrence of hv-CRKP is also being observed in other sequence types (STs), including ST15 and ST147 [21–24]. Therefore, this study aimed to find the risk factors for and mortality associated with CRKP infection by comparing the clinical characteristics of CRKP infection cases with those of CSKP infection cases and to identify the molecular characteristics, namely, the presence of carbapenem resistance genes (*bla*_KPC-2_, *bla*_NDM_ like and *bla*_OXA-48_ like) and four virulence genes (*rmpA*, *rmpA2*, *iroN* and *iucA*), that are correlated with the resistance and virulence of CRKP isolates.

## Methods

### Collection of bacterial isolates and medical records

The study was carried out in the 3300-bed Zhongnan Hospital of Wuhan University, a grade A tertiary general hospital in Hubei province. Three hundred and eleven non-duplicate isolates of *K. pneumoniae* were randomly collected from patients with *K. pneumoniae* infection admitted to this hospital from December 2019 to August 2022. These isolates were obtained from diverse clinical specimens, including sputum (*n* = 119, 38.3%), urine (*n* = 81, 26%), bronchoscopic lavage fluid (*n* = 32, 10.3%), blood (*n* = 27, 8.7%), drainage fluid (*n* = 18, 5.8%) and others fluids (ascites, bile, cerebrospinal fluid, hydrothorax secretion, perianal swab, pus, wound secretion and vaginal secretion) (*n* = 34, 10.9%).

Patients with *K. pneumoniae* infection were included in the study and classified as follows: *K. pneumoniae* infection cases were classified as bacteraemia (*n* = 27, 8.7%) when a blood culture was positive for a *K. pneumoniae* strain and the patient had clinical signs of systemic inflammatory response syndrome; *K. pneumoniae* infections were classified as nonbacteraemia through nonblood cultures, typically for cases such as pneumonia (*n* = 151, 48.5%), urinary tract infection (*n* = 81, 26%), liver abscess (*n* = 9, 2.8%), abdominal infection (*n* = 16, 5.1%), cholecystitis (*n* = 8, 2.6%) and others (*n* = 19, 6.1%). Electronic medical records of the patients were retrospectively collected, and the following data were extracted: gender; age; smoking status; comorbidities such as diabetes, hypertension, liver abscess, respiratory failure, cancer (including haematological malignancies) and neurological disorders, use of antibiotics in the 3 months before the current hospitalisation and during the current hospitalisation until a positive *K. pneumoniae* culture was obtained; initial severity (in the ICU); immunosuppressive conditions containing immunosuppressives therapy, the carry of human immunodeficiency virus (HIV) and high dosage administration of corticoids; the coexistence of carbapenem-resistant *Acinetobacter baumannii* in the clinical specimen and mortality outcome of this hospitalisation. This study was approved by the Ethics Committee of Zhongnan Hospital, Wuhan University.

### *K. pneumoniae* isolate identification and antimicrobial susceptibility testing (AST)

All *K. pneumoniae* isolates were identified using matrix-assisted laser desorption/ionisation time-of-flight (MALDI-TOF) mass spectrometry (Vitek MS, bioMérieux). The AST for the *K. pneumoniae* isolates was carried out using a VITEK 2 Compact System and the VITEK 2 AST-GN16 Test Kit (bioMerieux Inc., Durham, NC, USA), which contains the following antibiotics: amikacin (AN), amoxicillin–clavulanicn acid (AMC), ampicillin (AM), aztreonam (ATM), cefazolin (CZ), cefepime (FEP), cefoxitin (FOX), ceftriaxone (CRO), ciprofloxacin (CIP), gentamicin (GM), imipenem (IPM), levofloxacin (LEV), piperacillin–tazobactam (TZP), tobramycin (TM) and trimethoprim–sulfamethoxazole (SXT). In addition, the Kirby–Bauer method was used to test the ceftazidime–avibactam (CZA) susceptibility of the CRKP isolates according to the manufacturer’s instructions. Antibiotic susceptibility was defined according to the antibiotic breakpoints established by the Clinical & Laboratory Standards Institute. Following the European Committee on Antimicrobial Susceptibility Testing (EUCAST) guidelines, the minimum inhibitory concentrations (MICs) of polymyxin B for CRKP strains were determined by the broth microdilution method, and the breakpoints for colistin were as reported by EUCAST (breakpoint tables for the interpretation of MICs and zone diameters, Version 11.0).

### Detection of antimicrobial resistance and virulence genes

Genomic DNA was extracted from the *K. pneumoniae* isolates using a DNA extraction kit (Aidlab, Beijing) according to the manufacturer’s instructions. All primers used in the present study are shown in Table 1. The presence of three carbapenemase genes, *bla*_KPC-2_, *bla*_NDM_ like and *bla*_OXA-48_ like, in the CRKP isolates was tested using a polymerase chain reaction (PCR) assay. The final volume of the PCR reaction mixture was 25 µL, which included 12.5 µL of 2 × buffer (Aidlab, Beijing), 9.5 µL ddH_2_O, 1 µL forward primer (10 µM), 1 µL reverse primer (10 µM) and 1 µL genomic DNA. The PCR amplification conditions were as follows: denaturation at 95 °C for 3 min, followed by 42 cycles of 95 °C for 30 s, 57 °C for 20 s and 72 °C for 20 s and final extension at 72 °C for 5 min. The PCR products were then subjected to 1.5–2.0% agarose gel electrophoresis for detection. The primers of the virulence genes *rmpA*, *rmpA2*, *iroN* and *iucA* were those used in a previous study [20]. The annealing temperature and elongation time were determined according to the primer and product length.

**Table 1:** Primers used to screen for the presence of *bla*_KPC-2_, *bla*_NDM_ like and *bla*_OXA-48_ like in *K. pneumoniae*

Target genes	Primer name	Primer sequence	Product size	Reference
*bla*_KPC-2_	*bla*_KPC-2_-F	ATGCGCTCTATCGGCGATAC	91 bp	This study
	*bla*_KPC-2_-R	ATGAGGTATCGCGCGCATC		
*bla*_NDM_ like	*bla*_NDM_ like-F	GGGCCGTATGAGTGATTGCG	213 bp	This study
	*bla*_NDM_ like-R	ATATCACCGTTGGGATCGAC		
*bla*_OXA-48_ like	*bla*_OXA-48_ like -F	TTAAGTGGGATGGACAGACG	179 bp	This study
	*bla*_OXA-48_ like-R	ATTGCCCGAAATGTCCTCAT		

### Multi-locus sequence typing (MLST)

Seven house-keeping genes (*infB*, *pgi*, *mdh*, *phoE*, *gapA*, *tonB* and *rpoB*) were amplified from the genomic DNA of the *K. pneumoniae* isolates for MLST as described previously [25]. The amplified products were purified and subjected to Sanger’s dideoxy DNA sequencing (Tianyi Huiyuan, China). The resulting sequences were compared with the existing *K. pneumoniae* gene sequences available on the Pasteur Institute MLST website (http://bigsdb.pasteur.fr/klebsiella/). Each clinical isolate was then classified by the ST.

### A *Galleria mellonella* model of *K. pneumoniae* infection

To test the virulence of the *K. pneumoniae* isolates, *G. mellonella* was purchased from Aidlab (China) and stored in the dark at 4 °C until testing. Overnight cultures of *K. pneumoniae* strains were washed with phosphate-buffered saline (PBS) and further adjusted with PBS to concentrations of 1 × 10^6^ CFU/mL. Each bacterial isolate was injected into 10 *G. mellonella*, and the survival rates of the *G. mellonella* were recorded. The experiment was repeated three times.

### Statistical analysis

SPSS 27.0 software was used for data processing. The risk factors for CRKP infection and infection-related mortality were analysed by univariate logistic regression analysis. The variables with *P* < 0.05 in the univariate analysis were included in the multivariate logistic regression model, and backward stepwise regression analysis was performed to identify independent risk factors. A chi-square test was used to analyse the co-occurrence of the four virulence genes in the CRKP and CSKP isolates. *P* < 0.05 was considered to indicate a statistically significant difference.

## Results

### Risk factors for CRKP infection

Factors associated with CRKP infection included prior use of two or more antibiotics (including carbapenems), prior use of carbapenems, hypertension, cancer, neurological disorders, ICU admission, and the coexistence of carbapenem-resistant *A. baumannii* in clinical specimens (*P* < 0.05) (Table 2). Among them, hypertension (odds ratio [OR], 1.824; 95% confidence interval [CI], 1.053–3.161; *P* = 0.032), neurological disorders (OR, 2.890; 95% CI, 1.568–5.326; *P* < 0.001), ICU admission (OR, 2.099; 95% CI, 1.148–3.836; *P* = 0.016), prior use of two or more antibiotics (OR, 4.124; 95% CI, 2.142–7.939; *P* < 0.001) and prior use of carbapenems (OR, 2.613; 95% CI, 1.314–5.196; *P* = 0.006) were independent risk factors for CRKP infection (Table 2).

**Table 2:** Clinical characteristics of patients infected with *K. pneumoniae*

Risk factors	Variables		Univariate analysis	Multivariate analysis	
	CRKP (*n* = 140)	CSKP (*n* = 171)	*P*	OR (95% CI)	*P*
Age (years)	63.6 $\pm$ 18.7	63.7 $\pm$ 16.3	0.949		
Male, *n* (%)	91 (65.0%)	120 (70.2%)	0.333		
Smoking	30 (21.4%)	41 (24.0%)	0.596		
Comorbidities, *n* (%)					
Diabetes	45 (32.1%)	51 (29.8%)	0.661		
Hypertension	73 (52.1%)	70 (40.9%)	0.049	1.824 (1.053–3.161)	0.032
Liver abscess	4 (2.9%)	12 (7.0%)	0.099		
Respiratory failure	31 (22.1%)	25 (14.6%)	0.086		
Cancer	19 (13.6%)	40(23.4%)	0.028	1.163 (0.545–2.480)	0.696
Neurological disorders	78 (55.7%)	46 (26.9%)	< 0.001	2.890 (1.568–5.326)	< 0.001
Initial severity, *n* (%)					
ICU	97 (69.3%)	63 (36.8%)	< 0.001	2.099 (1.148–3.836)	0.016
Immunosuppressive conditions, *n* (%)	36 (25.9%)	51 (29.8%)	0.446		
Antibiotic use before *K. pneumoniae* infection, *n* (%)					
Two or more antibiotics	113 (80.7%)	56(32.7%)	< 0.001	4.124 (2.142–7.939)	< 0.001
Carbapenem antibiotic	71 (50.7%)	28 (16.4%)	< 0.001	2.613 (1.314–5.196)	0.006
The coexistence of carbapenem-resistant *A. baumannii* in clinical specimens, *n* (%)	28 (20.0%)	16 (9.4%)	0.007	0.695 (0.313–1.541)	0.370
Outcomes Death, *n* (%)	33 (23.6%)	24 (14.0%)	0.031		

*OR*: odds ratio; *CI*: confidence interval; *ICU*: intensive care unit. The variables with* P* < 0.05 screened in univariate analysis were included in multivariate logistic regression analysis

### Clinical cohort study of mortality: survival versus death in patients with *K. pneumoniae* infection

Among the 311 patients with *K. pneumoniae* infection, 57 (18.3%) patients died. Univariate analysis showed that respiratory failure, ICU admission, and prior use of carbapenems were factors related to death of in these patients. Multivariate analysis confirmed that respiratory failure (OR, 5.909; 95% CI, 2.911–11.996; *P* < 0.001) and ICU admission (OR, 12.285; 95% CI, 4.180–36.109; *P* < 0.001) were independent risk factors for death in these patients (Table 3).

**Table 3:** Analysis of associated risk factors for death associated with *K. pneumoniae* infection

Risk factors	Variables		Univariate analysis	Multivariate analysis	
	Death (*n* = 57)	Survival (*n* = 254)	*P*	OR (95% CI)	*P*
Age (years)	65.9 ± 18.8	63.1 ± 17.1	0.265		
Male, *n* (%)	37 (64.9%)	174 (68.5%)	0.601		
Smoking	9 (15.8%)	62 (24.4%)	0.162		
Accompanying disease, *n* (%)					
Diabetes	18 (31.6%)	78 (30.7%)	0.898		
Hypertension	27 (47.4%)	116 (45.7%)	0.817		
Liver abscess	1 (1.8%)	15 (5.9%)	0.201		
Respiratory failure	30 (52.6%)	26 (10.2%)	< 0.001	5.909 (2.911–11.996)	< 0.001
Cancer	8 (14.0%)	51 (20.1%)	0.294		
Neurological disorders	25 (43.9%)	99 (39.0%)	0.498		
Initial severity, *n* (%)					
ICU	53 (93.0%)	107 (42.1%)	< 0.001	12.285 (4.180–36.109)	< 0.001
Immunosuppressive conditions, *n* (%)	19 (33.3%)	68 (26.9%)	0.329		
Antibiotic use before *K. pneumoniae* infection, *n* (%)					
Two or more antibiotics	37 (64.9%)	132 (52.0%)	0.077		
Carbapenem antibiotic	26 (45.6%)	73 (28.7%)	0.013	1.124 (0.564–2.239)	0.739
The coexistence of carbapenem-resistant *A. baumannii* in clinical specimens (%)	12 (21.1%)	32 (12.6%)	0.099		
Co-carry *rmpA*, *rmpA2*, *iroN* and *iucA*, *n* (%)	25 (43.9%)	108 (42.5%)	0.854		

### AST of the *K. pneumoniae* isolates

All of the *K. pneumoniae* isolates were resistant to AM. In total, 140 (45.0%) isolates were categorised as CRKP based on their IPM susceptibility. Compared with the CSKP isolates, the CRKP isolates had higher resistance rates to all antibiotics except ampicillin, to which all isolates were resistant. In addition, the CRKP isolates showed resistance rates of 1.4% and 6.4% for polymyxin B and ceftazidime–avibactam, respectively (Table 4). The detailed results of antibiotic susceptibility testing of CSKP and CRKP isolates are presented in Table 4.

**Table 4:** Antimicrobial resistance profiles of *K. pneumoniae*

	All *K. pneumoniae* (*n* = 311)	CRKP (*n* = 140)	CSKP (*n* = 171)	*P* (comparison between CRKP and CSKP)
Antibiotics	Resistance	Resistance	Resistance	
IPM, *n* (%)	140 (45.0)	140 (100.0)	0 (0.0)	/
AM	311 (100.0)	140 (100.0)	171 (100.0)	/
AN	115 (37.0)	114 (81.4)	1 (0.6)	< 0.001
ATM	173 (55.6)	134 (95.7)	39(22.8)	< 0.001
AMC	155 (49.8)	135 (96.4)	20 (11.7)	< 0.001
CZ	218 (70.1)	137 (97.9)	81(47.4)	< 0.001
CRO	184 (59.2)	135 (96.4)	49 (28.7)	< 0.001
CIP	193 (62.1)	136 (97.1)	57 (33.3)	< 0.001
FOX	154(50.0)	133 (95.0)	21 (12.3)	< 0.001
FEP	181(58.2)	134 (95.7)	47 (27.5)	< 0.001
GM	140(45.0)	122 (87.1)	18 (10.5)	< 0.001
LEV	179(57.6)	136 (97.1)	43 (25.1)	< 0.001
SXT	154(49.5)	113 (80.7)	41 (24.0)	< 0.001
TZP	141(45.3)	135 (96.4)	6 (3.5)	< 0.001
TM	128(41.2)	119 (85.0)	9 (5.3)	< 0.001
PB	/	2 (1.4)	/	/
CZA	/	9 (6.4)	/	/

*AM*: ampicillin; *AN*: amikacin; *ATM*: aztreonam; *AMC*: amoxicillin-clavulanicn acid; cefazolin; ceftriaxone; *CIP*: ciprofloxacin; *FOX*: cefoxitin; *FEP*: cefepime; *GM*: gentamicin; *LEV*: levofloxacin; *SXT*: trimethoprim/sulfamethoxazole; *TZP*: piperacillin/tazobactam; tobramycin; *PB*: polymyxin B; *CZA*: ceftazidime-avibactam

### MLST of the *K. pneumoniae* isolates

**Fig. 1:**
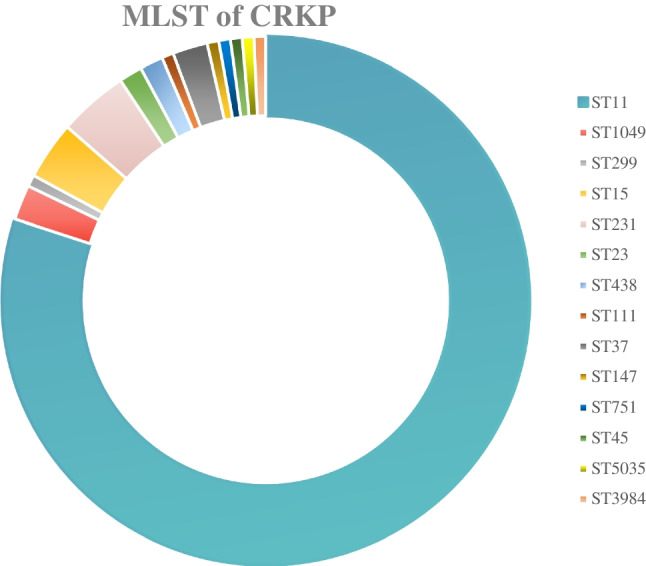
Classification characteristics of MLST of CRKP

All isolates were successfully typed by MLST and assigned to 102 STs. The most prevalent ST was ST11 (112, 36.0%), followed by ST23 (15, 4.8%), ST37 (9, 2.9%) and ST15 (9, 2.9%). Notably, all of the 112 ST11 isolates were CRKP. Fourteen STs were found among the 140 CRKP isolates. The detailed results of MLST for CRKP isolates are provided in Fig. 1. Among the CSKP isolates, 95 STs were found, with ST23 (13, 7.6%) being the most common ST, followed by ST68 (8, 4.7%), ST37 (6, 3.5%), ST25 (5, 2.9%), ST15 (4, 2.3%), ST412 (4, 2.3%), ST36 (4, 2.3%) and ST307 (4, 2.3%).

### Virulence gene and resistance gene profiles

Three hundred and eleven *K. pneumoniae* isolates were tested for the presence of the virulence genes *rmpA*, *rmpA2*, *iroN* and *iucA*. Overall, the positive rates of *rmpA*, *rmpA2*, *iroN* and *iucA* were 58.2% (181), 48.6% (151), 51.1% (159) and 46.6% (145), respectively. Furthermore, 133 (42.8%) of the total isolates contained all of the four virulence genes, 53 (17.0%) isolates contained one to three of the virulence genes, whereas 125 (40.2%) isolates carried none of the virulence genes. Among the 140 CRKP isolates, the positive rates of *rmpA*, *rmpA2*, *iroN* and *iucA* were 67.1% (94), 62.9% (88), 60.0% (84) and 59.3% (83), respectively; 83 (59.3%) of these isolates contained all of the four virulence genes, 13 (0.9%) isolates contained one to three of the virulence genes, while 44 (31.4%) isolates carried none of the virulence genes. Among the 171 CSKP isolates, the positive rates of *rmpA*, *rmpA2*, *iroN* and *iucA* were 50.9% (87), 36.8% (63), 43.9% (75) and 36.3% (62) respectively; 50 (29.2%) of these isolates contained all of the four virulence genes, 40 (23.4%) isolates contained one to three of the virulence genes, while 81 (47.4%) isolates carried none of the virulence genes. As shown in Table 5, CRKP was more likely than CSKP to carry all of the four virulence genes.

**Table 5:** Analysis of *K. pneumoniae* carrying all of the four virulence genes

Virulence genes	CRKP (*n* = 140)	CSKP (*n* = 171)	*P*
Co-carry *rmpA*, *rmpA2*, *iroN* and *iucA*, *n* (%)	83 (59.3%)	50 (29.2%)	< 0.001

**Fig. 2:**
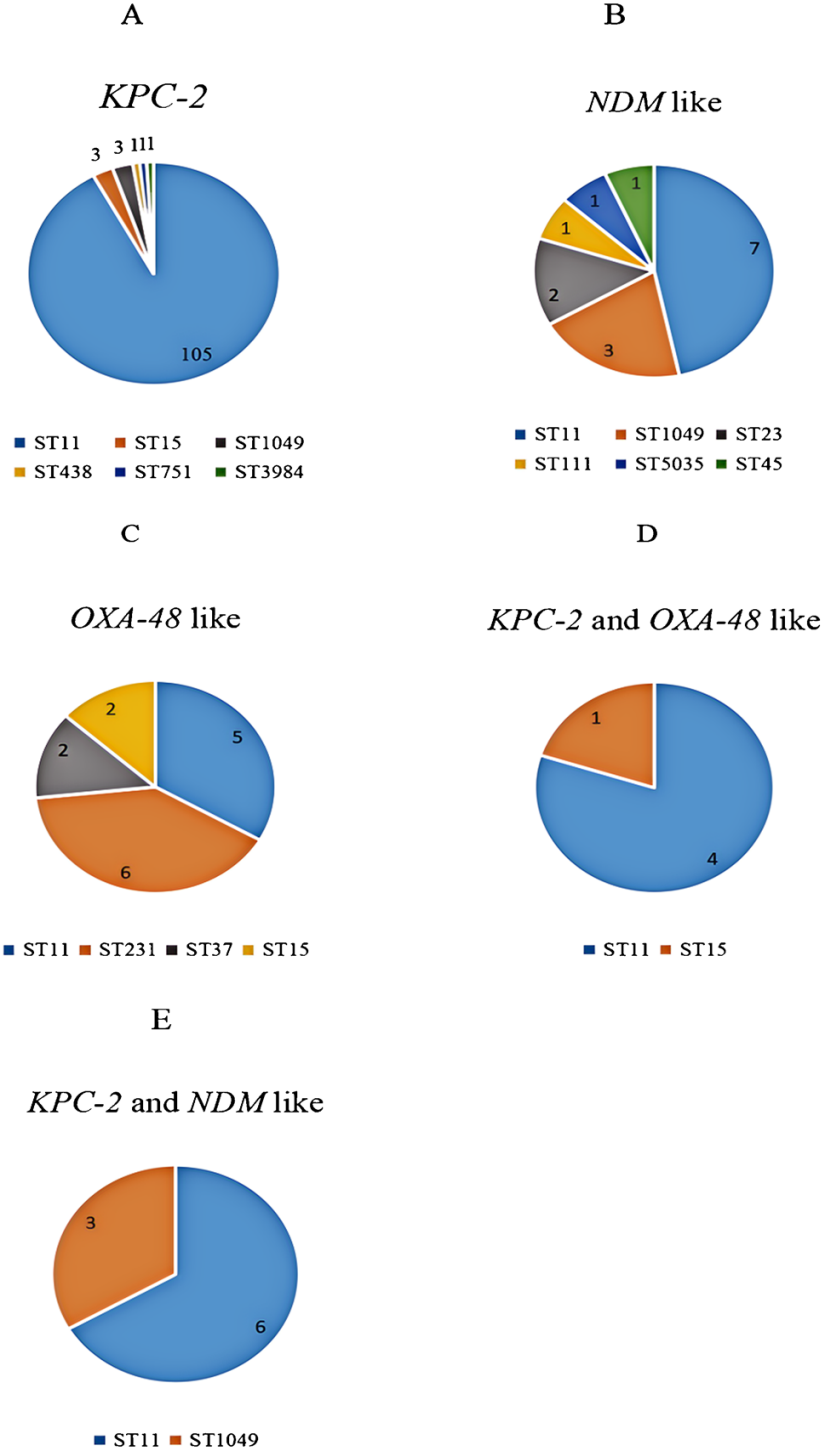
Genotyping characteristics of various drug resistance genes of CRKP

**Fig. 3:**
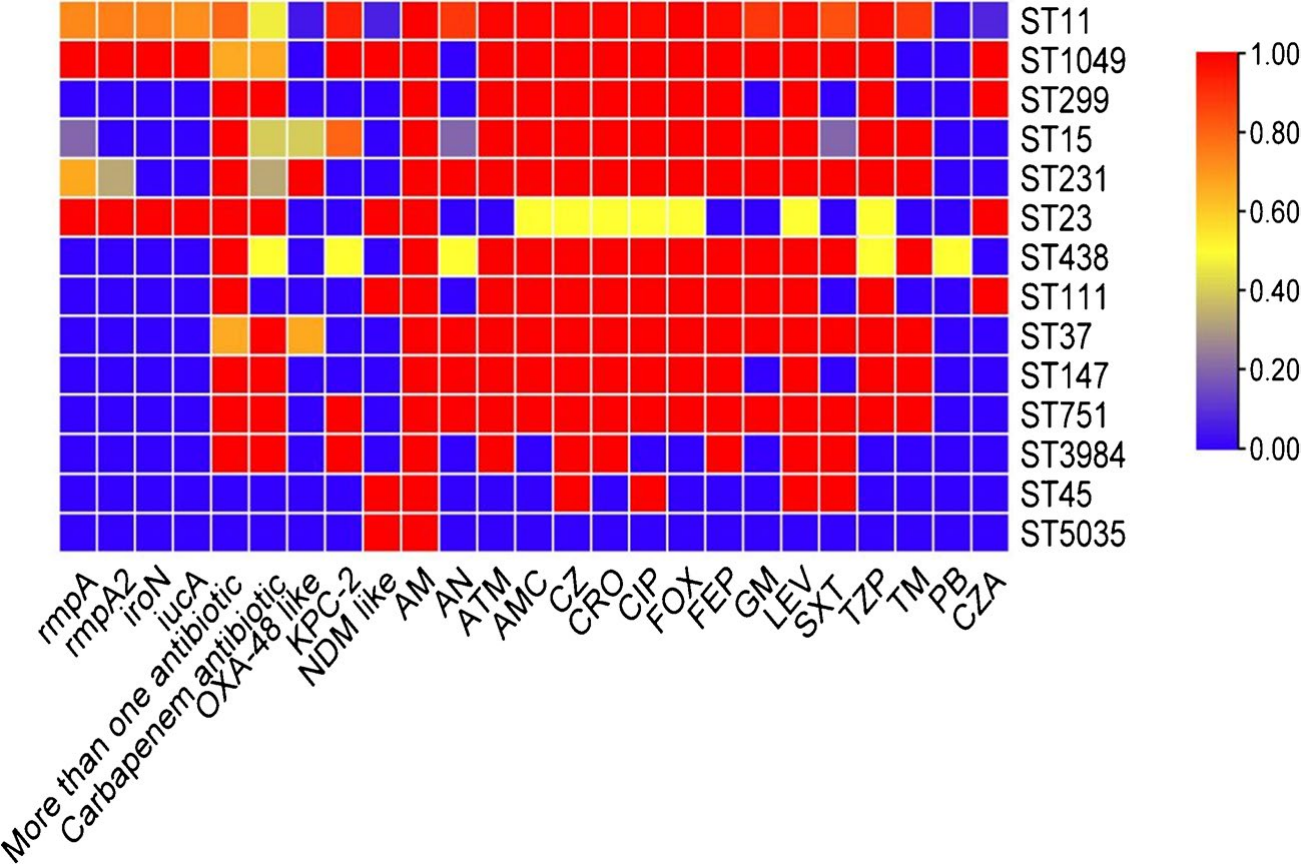
Heatmap of virulence gene carrier rates, multiple antibiotic use rates, carbapenem antibiotic use rates and antimicrobial resistance rates in different MLST CRKP strains

Three common carbapenemase-encoding genes—*bla*_KPC-2_, *bla*_NDM_ like and *bla*_OXA-48_ like—were detected using a PCR assay. Among the 140 CRKP isolates, the positive rates of *bla*_KPC-2_, *bla*_NDM_ like and *bla*_OXA-48_ like were 82.1%, 10.7% and 10.7%, respectively. The distribution of resistance genes in different STs is shown in Fig. 2. Of the 112 ST11 isolates, 105 (93.8%) harboured *bla*_KPC-2_; moreover, 78 (74.3%) of these 105 ST11-*KPC-2* isolates contained all of the four virulence genes *rmpA*, *rmpA2*, *iroN* and *iucA*. The proportional relationships between STs and prior antibiotic use, resistance genes, virulence genes, and resistance ratios to different antibiotics are shown in Fig. 3.

### Comparison of the G. mellonella virulence test

**Fig. 4:**
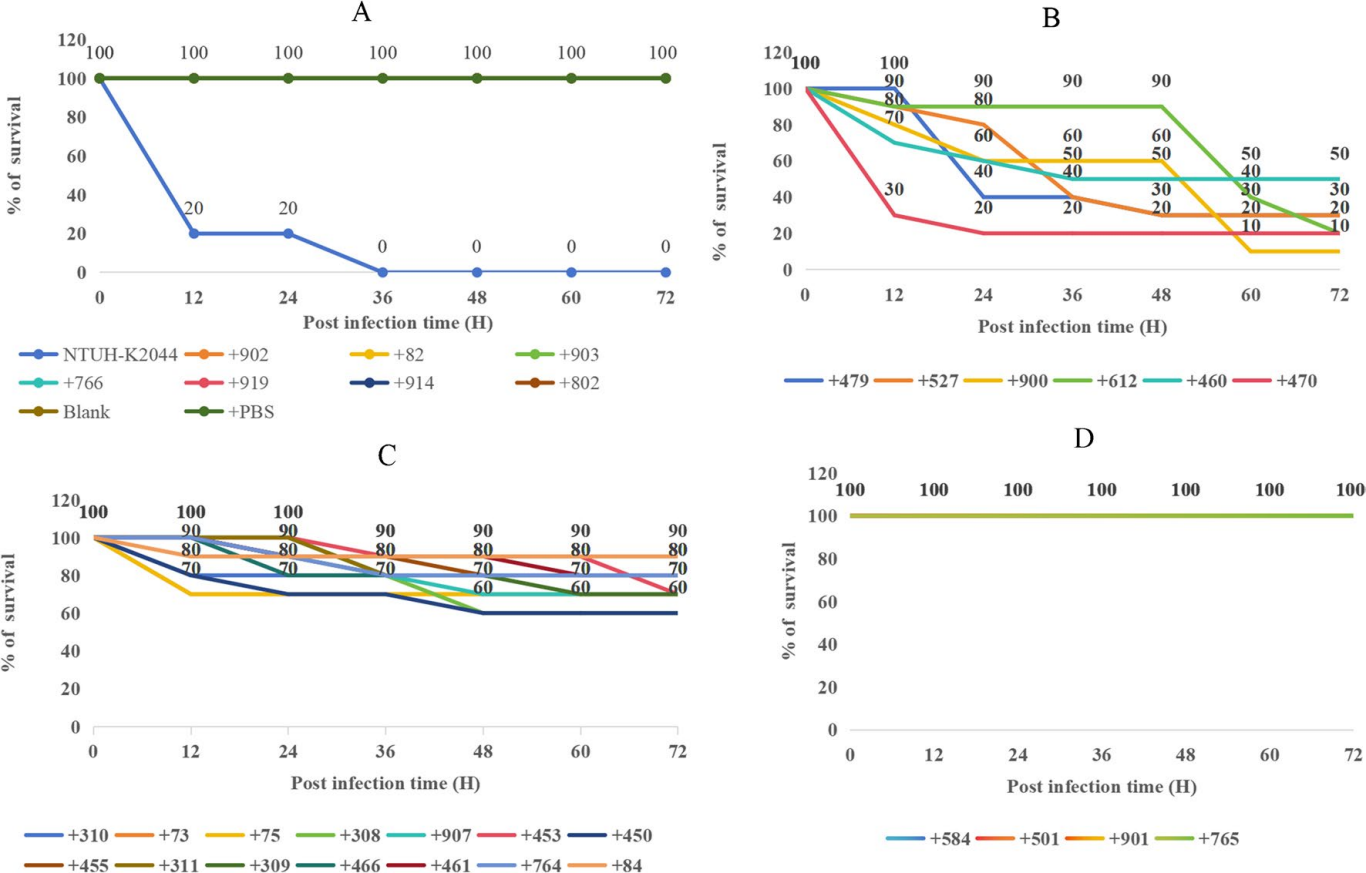
Experiments with different *K. pneumoniae* infecting *Galleria mellonella*. **A** Survival rates of NTUH-K2044 and 7 strains *K. pneumoniae* without these four virulence genes, + PBS empty control and blank control. Survival rates of 24 strains containing these four virulence genes; **B** 6 strains were high virulent, with a survival rate of 50% or less within 72 h; **C** 14 strains showed low virulence with a 72-h survival rate greater than 50%; **D** 4 strains were nonvirulent with 100% survival at 72 h. NTUH-K2044 carried these virulence genes: *iroB*, *iroC*, *iroD*, *iroN*, *iucA*, *iucB*, *iucC*, *iucD*, *iutA*, *rmpA* and *rmpA2*

Patients with CRKP infection had a higher mortality rate than those with CSKP infection (23.6% vs 14.0%,* P* < 0.05) (Table 2); however, co-carrying *rmpA*, *rmpA2*, *iroN* and *iucA* was not associated with mortality in patients with *K. pneumoniae* infection (43.9% vs 42.5%, *P* = 0.854) (Table 3). To further investigate this phenomenon, 24 isolates carrying all of the four virulence genes were randomly selected and subjected to the *G. mellonella* virulence test. It was found that six of these isolates exhibited high virulence with a 72-h *G. mellonella* survival rate < 50%, 14 isolates showed low virulence with a 72-h *G. mellonella* survival rate > 50%, and four isolates were found to be nonvirulent with a 72-h *G. mellonella* survival rate of 100%. The hypervirulent positive-control *K. pneumoniae* strain NTUH-K2044 was found to have a 36-h *G. mellonella* survival rate of 0%, whereas seven *K. pneumoniae* isolates without these four virulence genes and the negative control strain had a 72-h *G. mellonella* survival rate of 100% (Fig. 4).

## Discussion

To improve the efficacy of CRKP infection treatment, we investigated the risk factors for CRKP infection and found ICU admission and prior antibiotic use to be associated with CRKP infection, consistent with previous studies [26]. Hypertension has been reported to be associated with bloodstream infection of CRKP [27], and our study showed that it was also one of the risk factors for CRKP infection. *K. pneumoniae* and *A. baumannii* have a mutually beneficial relationship, protecting and feeding each other to promote their survival [28]. Plasmids carrying resistance genes can also be transferred between them [29]. Accordingly, we found that the coexistence of carbapenem-resistant *A. baumannii* was associated with CRKP infection. Our results found that patients with neurological disorders were more susceptible to CRKP infection than those without neurological disorders, which may be related to the former patients’ need for absolute bed rest. We also found that patients with cancer were more susceptible to CSKP infection than those without cancer, but the reason was not clear and needs to be explored in a further study.

Our results demonstrated that different STs tended to carry different resistance genes. As in many other studies in China, a high clonal diversity of *K. pneumoniae* was observed in our study, with ST11 being the predominant ST [8, 30–32]. Another important phenomenon observed was that all of the ST11 isolates were CRKP with a *bla*_KPC-2_ positive rate of 93.8%, and ST11 isolates harbouring *bla*_KPC-2_ (ST11-*KPC-2*) accounted for 80.0% of the CRKP isolates and 36.0% of the total *K. pneumoniae* isolates. This suggests that ST11-*KPC-2* was responsible for most of the *K. pneumoniae* infection cases. Thus, there is an urgent need to prevent and control the further spread of ST11-*KPC-2* in hospitals across China. Furthermore, this study was the first to identify ST438, ST751 and ST3984 carrying *bla*_KPC-2_; ST111 and ST5035 carrying *bla*_NDM_ like; and ST1049 carrying both *bla*_KPC-2_ and *bla*_NDM_ like.

Infection cases attributable to hv-CRKP isolates are increasing by the year in hospitals, and plasmid recombination and fusion events have been found to be the two important evolutionary pathways responsible for the emergence of hv-CRKP isolates [22, 33]. The virulence plasmid pLVPK containing two capsular polysaccharide regulator genes (*rmpA* and *rmpA2*) and several siderophore gene clusters (e.g. *iucABCD*/*iutA*/*iroBCDN* clusters) is thought to contribute to the hypermucoviscous phenotype of *K. pneumoniae* [20, 18, 19]. Therefore, we further investigated the presence of four representative virulence genes carried by pLVPK in our isolates*.* We found that 133 (42.8%) of all *K. pneumoniae* isolates and 83 (59.3%) of the CRKP isolates carried all of the four virulence genes *rmpA*, *rmpA2*, *iroN* and *iucA*, and 78 of those 83 (94.0%) CRKP strains belonged to ST11-*KPC-2*. Such high proportions of *K. pneumoniae*, CRKP and ST11-*KPC-2* isolates carrying all of the four virulence genes have not been previously reported. A study reported that among 1052 CRKP strains isolated during 2015–2017 from 56 centres across China, only 72 (6.8%) strains carried all of the four virulence genes [34]. In recent years, the highest prevalence of a single virulence gene in CRKP isolates was approximately 20% in China and Vietnam, followed by 10% in Qatar, and less than 10% in the USA and the UK [21, 35–37]. Thus, the mechanism underlying the simultaneous presence of all four of these virulence genes at high rates in *K. pneumoniae*, CRKP and ST11-*KPC-2* isolates is currently unknown and needs to be investigated in the future.

In *K. pneumoniae*, the occurrence of pLVPK or pLVPK-like virulent plasmids was reported to have a strong correlation with highly hypervirulent phenotypes. Several experiments have confirmed the hypervirulent phenotype of pLVPK-positive isolates, including the string test, serum killing assay and the *G. mellonella* infection model [20, 22]. Highly hypervirulent phenotypes of *K. pneumoniae* have been reported to be associated with high mortality rates [38–41]. However, in the present study, carbapenem resistance rather than the co-occurrence of the virulence genes *rmpA*, *rmpA2*, *iroN* and *iucA* in the isolates was associated with patient death during hospitalisation. The possible reasons for the inconsistency include low expression levels of virulence genes under host immune pressure, prior use of two or more antibiotics or carbapenems, serious underlying diseases and a small sample size in our study. Supporting our speculation, only a small proportion of the *K. pneumoniae* isolates containing these four virulence genes was found to exhibit high virulence in the *G. mellonella* infection model.

Given our study’s limitations of being a single-centre study and having a small sample size, a multicentre study with a large sample size is warranted to confirm our results.

In conclusion, the independent risk factors for CRKP infection in our hospital were diverse. ST11-*KPC-2*, which carried all of the four virulence genes, was responsible for most of the *K. pneumoniae* infection cases. Furthermore, patient mortality was associated with carbapenem resistance rather than with the co-occurrence of all of the four virulence genes.

## Data Availability

Not applicable.
